# A conserved hexanucleotide motif is important in UV-inducible promoters in *Sulfolobus acidocaldarius*

**DOI:** 10.1099/mic.0.000455

**Published:** 2017-05-03

**Authors:** Thuong Ngoc Le, Alexander Wagner, Sonja-Verena Albers

**Affiliations:** Molecular Biology of Archaea, Institute of Biology II - Microbiology, University of Freiburg, Schaenzlestrasse 1, 79104 Freiburg, Germany

**Keywords:** ups pili, archaea, *Sulfolobus*, regulation, promoter, tfb3

## Abstract

Upon DNA damage, Sulfolobales exhibit a global gene regulatory response resulting in the expression of DNA transfer and repair proteins and the repression of the cell division machinery. Because the archaeal DNA damage response is still poorly understood, we investigated the promoters of the highly induced *ups* operon. Ups pili are involved in cellular aggregation and DNA exchange between cells. With LacS reporter gene assays we identified a conserved, non-palindromic hexanucleotide motif upstream of the *ups* core promoter elements to be essential for promoter activity. Substitution of this cis regulatory motif in the *ups* promoters resulted in abolishment of cellular aggregation and reduced DNA transfer. By screening the *Sulfolobus acidocaldarius* genome we identified a total of 214 genes harbouring the hexanucleotide motif in their respective promoter regions. Many of these genes were previously found to be regulated upon UV light treatment. Given the fact that the identified motif is conserved among *S. acidocaldarius* and *Sulfolobus tokodaii* promoters, we speculate that a common regulatory mechanism is present in these two species in response to DNA-damaging conditions.

## Introduction

The transcription machinery of archaea resembles a simplified version of the eukaryotic RNA polymerase (RNAP) II system, while the mechanisms of transcriptional regulation are more related to bacteria [1–6]. The canonical core promoter of archaea consists of a TATA box, an AT-rich region located around −26 to −30 bp upstream from the transcription start site (TSS). Directly upstream of the TATA box is the transcription factor B recognition element (BRE) [1, 7]. These core elements are recognized by the TATA binding protein (TBP) and transcription factor B (TFB), respectively. Binding of these general transcription factors to the promoter subsequently recruits the RNAP to the TSS, thereby forming the pre-initiation complex (PIC) [8–12].

The transcription of genes is regulated through the binding of regulatory proteins to a specific sequence in the promoter region. Usually, the location of the binding site with respect to the promoter determines whether the transcription factor functions as an activator or repressor [13]. Archaeal repressors, for instance, usually bind to sites which overlap or are downstream of the core promoter. Thereby the repressor can prevent the binding of the TBP, TFB or RNAP to the promoter, which consequently leads to inhibition of PIC formation [14–18]. While archaeal repression is quite well understood, the *bona fide* mechanisms of gene activation in archaea remains under-investigated. To date, it is known that binding of activators upstream of the core promoter either works by supporting the binding of TBP and TFB to the promoter sequences or by stabilizing the PIC [19–21]. To date, a few DNA regulatory sequences were reported in archaea. These include some activating sequences (the ARA box, the ArnR box 1, the ss-LrpB binding site), repressing motifs (the binding site of Phr, TrmB) and the recognition motif of the global regulator TrmBL1 [16, 18, 22–26]. These DNA sequence motifs are semi-palindromic sequences with a number of less- or non-conserved base pairs in the centre [18, 22, 27].

*Sulfolobus acidocaldarius* is a Crenarchaeon thriving in extreme habitats like terrestrial solfataric hot springs, where environmental stresses such as high temperature, pH and UV irradiation are a constant threat to the genome integrity of the cells [28]. Thus, *S. acidocaldarius* is an ideal model organism to study the poorly understood regulation in response to environmental stress in archaea. Microarray studies on *Sulfolobus* species in response to UV stress showed a clear transcriptional response. These data revealed the repression of DNA replication and chromatin proteins, the upregulation of beta-carotene biosynthetic enzymes, proteins that detoxify reactive oxygen species and proteins which recently were described as functioning in importing DNA [29–31]. One of the highest upregulated operons is responsible for the biogenesis of a type IV pilus – the ups pilus (UV-inducible pilus of Sulfolobales). Ups pili are responsible for cellular aggregation and subsequent DNA exchange between cells [29, 32]. The *ups* operon encodes a protein of unknown function (UpsX), an ATPase (UpsE), a membrane protein (UpsF) and two pre-pilin subunits (UpsA and UpsB) [32–34] Except for UpsX, all proteins encoded by the *ups* operon are essential for pili formation and cellular aggregation upon UV stress [34]. To date, the function of UpsX is not clear, but a deletion mutant of *upsX* showed a significant reduction of DNA transfer [34].

Previous deep sequencing studies on *S. acidocaldarius* cDNA suggest a primary TSS in front of *upsX* and secondary TSSs in front of *upsE* and *upsA* [35]. However, it was unclear how the operon is regulated in response to UV irradiation. In our study, we identified for the first time a conserved, cis regulatory motif in promoters of genes that are regulated after UV stress. This motif was shown to be essential for the activity of the *ups* operon and consequently in cellular aggregation and DNA transfer in *S. acidocaldarius*.

## Methods

### Growth conditions

*S. acidocaldarius* strains (Table 1) were grown aerobically at 75 °C in basal Brock medium [28] at pH 3.5, supplemented with 0.1 % NZ-amine, 0.2 % dextrin and 20 µg ml^−1^ uracil. Plasmid-containing strains were grown in the same medium but without the addition of uracil. For cultivation on solid media, 1.2 % gelrite was added to the Brock solution. Plates were incubated for 5–7 days at 75 °C. *E. coli* strains, Top10 and ER1821 (NEB), used for cloning and methylation of plasmid DNA, respectively, were grown in Lysogeny broth medium (10 g l^−1^ tryptone, 5 g l^−1^ yeast extract, 10 g l^−1^ NaCl) at 37 °C supplemented with the appropriate antibiotics. The growth of the cells was monitored by measurement of the optical density at 600 nm (OD_600_).

**Table 1.** Strains and plasmids

	Strain	Background strain	Genotype	Reference
1	MW001	*S. acidocaldarius* DSM639	∆*pyrE* (∆bp 91–412)	[37]
2	JDS22	*S. acidocaldarius* DSM639	∆*pyrE* (∆bp 16–38)	[53]
3	MW109	*S. acidocaldarius* MW001	∆*upsE*	[37]
4	MW115	*S. acidocaldarius* MW001	∆*upsX*	[34]
5	MW1100	*S. acidocaldarius* MW001	∆*upsX* (∆bp −371–1873)	This study
6	MW1101	*S. acidocaldarius* MW001	Substitution on *upsX* promoter (ATTT>cggg)	This study
7	MW1103	*S. acidocaldarius* MW001	Substitution on *upsE* promoter (ACTTTC>caggga)	This study
	**Plasmids**	**Backbone plasmid**	**Description**	
1	pCMalLacS		Backbone for mutated promoters	[36]
2	pSVA406		Backbone for in frame deletion plasmids	[37]
3	pSVA3600	pCMalLacS	P*malE*> P*upsX* (339 bp)	This study
4	pSVA3601	pCMalLacS	P*malE*> P*upsX* (193 bp)	This study
5	pSVA3602	pCMalLacS	P*malE*> P*upsX* (97 bp)	This study
6	pSVA3603	pCMalLacS	P*malE*> P*upsX* (46 bp)	This study
7	pSVA3606	pCMalLacS	P*malE*> P*upsX* (39 bp)	This study
8	pSVA3610	pSVA3600	P*upsX* sub-46 GT>tg	This study
9	pSVA3611	pSVA3600	P*upsX* sub-44 AT>cg	This study
10	pSVA3612	pSVA3600	P*upsX* sub-42TT>gg	This study
11	pSVA3613	pSVA3600	P*upsX* sub-40 TC>ga	This study
12	pSVA3631	pSVA406	P*malE*> P*upsE* (350 bp)	This study
13	pSVA3632	pSVA3631	Substitution on *upsE* promoter (ACTTTC>caggga)	This study
14	pSVA3622	pSVA406	KO plasmid ∆ 1873 *upsX*+321 bp *upsX* promoter	This study
15	pSVA3625	pSVA406	Substitution plasmid P *upsX* (ATTT>cggg)	This study
16	pSVA3648	pSVA406	Substitution plasmid P *upsE* (ACTTTC>caggga)	This study

### UV treatment and aggregation assays

UV light exposure of *S. acidocaldarius* cells was performed as described previously [30]. Ten millilitres of culture (OD_600_ 0.2–0.3) was induced with 75 J m^−^² UV light (254 nm, Spectroline UV-crosslinker) in a Petri dish. After that, cultures were incubated at 75 °C for 3 h. Subsequently, 5 ml of each culture (diluted to OD_600_ 0.2) was spotted on a microscope slide coated with 1 % agarose. Single and aggregated cells (*n*>3) were analysed by an ImageJ cell counter (NIH, Bethesda, MD) from at least three independent experiments. The percentage of cells found in aggregates was subsequently calculated.

### Construction of mutated *upsX* promoter sequences

The plasmid pcMalLacS [36] containing LacS under control of the *malE* promoter was used as a backbone plasmid. In this study, the *malE* promoter was replaced by the respective *upsX (Saci_1493)* promoters (Table 1). Truncated promoters were amplified from genomic *S. acidocaldarius* DNA by specific forward and reverse primers (Table 2). After treatment with SacII and NcoI, promoter truncations were ligated into pcMalLacS treated with the same enzymes, resulting in the plasmids listed in Table 1.

**Table 2.** Primers

No	Name	Sequence	Description	Reference
1	5504	TATCCGCGGAAGAGAAGCCTTGGATTG	fw *upsX* promoter 339 bp	This study
2	5505	TTACCATGGATTTCTTCGATGCTGTAAAATATACAG	rv *upsX* promoter 339 bp	This study
3	5506	TATCCGCGGAATATCAAAACATATCATTAAAGC	fw *upsX* promoter 193 bp	This study
4	5507	TATCCGCGGTATGGTAACCACATATAATCTCC	fw *upsX* promoter 97 bp	This study
5	5508	TATCCGCGGTATTTTCAAACCACACTCTCTG	fw *upsX* promoter 46 bp	This study
6	5515	TATCCGCGGCAAACCACACTCTCTGTATATTTTACAGCATCGAAGAAATAACCATGGTTA	fw *upsX* promoter 39 bp	This study
7	5516	TAACCATGGTTATTTCTTCGATGCTGTAAAATATACAGAGAGTGTGGTTTGCCGCGGATA	rv *upsX* promoter 39 bp	This study
8	5523	TGAAAATCATATTAACGAGATAGGCA	rv *upsX* 339 bp Sub −46 GT/tg	This study
9	5524	TCGTTAATATGATTTTCAAACCACAC	fw *upsX* 339 bp Sub −46 GT/tg	This study
10	5525	TGAAACGACTATTAACGAGATAGGCA	rv *upsX* 339 bp sub −44 AT/cg	This study
11	5526	TCGTTAATAGTCGTTTCAAACCACAC	fw *upsX* 339 bp sub −44 AT/cg	This study
12	5527	TGACCATACTATTAACGAGATAGGCA	rv *upsX* 339 bp sub −42 TT/gg	This study
13	5528	TCGTTAATAGTATGGTCAAACCACAC	fw *upsX* 339 bp sub −42 TT/gg	This study
14	5529	TTTTCAAATACTATTAACGAGATAGGCAATA	rv *upsX* 339 bp sub −40 TC/ga	This study
15	5530	TCGTTAATAGTATTTGAAAACCACACTCT	fw *upsX* 339 bp sub −40 TC/ga	This study
16	5553	TGGGTCTATGCAATCCAAGGCTTCTCTTCC	rv-ol-del 321 bp promoter +1783 bp ORF *upsX*	This study
17	5554	GCCTTGGATTGCATAGACCCAGCTTACAAC	fw-ol-del 321 bp promoter +1783 bp ORF *upsX*	This study
18	5563	GTGTGGTTTGACCCGACTATTAACGAG	rv ol sub −44ATTT/cggg *upsX* promoter	This study
19	7543	TACAGGGAATTTTTCTGTCTGTCTTTTAATATTTC	rv ol sub −44ACTTTc/caggga *upsE* promoter	This study
20	7544	AAAAATTCCCTGTATTCAAGGAATAATTGCTGTGC	rv ol sub −44ACTTTc/caggga *upsE* promoter	This study
21	5564	GTCGGGTCAAACCACACTCTCTGTATATTTTAC	fw ol Sub −44ATTT/cggg *upsX* promoter	This study
22	5555	GAT*GGGCCC*TAGTCCCAGGTAGTAACTC	fw-US del 321 bp promoter +1783 bp ORF *upsX*	This study
23	5560	TATCTGCAGTATGACCCTCGGGCAATG	rv-DS del 321 bp promoter +1783 bp ORF *upsX*	This study
24	1905	CGTATTACCGCCTTTGAGTG	fw *upsX* promoter sequencing	

Substitutions in the *upsX* promoter were introduced by performing overlap PCR in the backbone plasmid pSVA3600 containing the full-length *upsX* promoter, by using specific overlap primer pairs (Table 2) and two outside primers, 1905 and 2946. Mutated fragments were subsequently cloned to the backbone plasmid, resulting in the plasmids listed in Table 1. Methylation and transformation of plasmids to MW001 were performed as described previously [37]. Single colonies were picked and transferred to Brock medium [28], pH 3.5, supplemented with 0.1 % NZ-amine and 0.2 % dextrin, for ONPG assay.

### ONPG assay

*S. acidocaldarius* plasmid-containing strains were grown in first-selection Brock medium (supplemented with 0.1 % NZ-amine and 0.2 % dextrin) for 2 days. Three biological replicates were inoculated from first-selection liquid cultures. Each was grown in 10 ml Brock with 0.1 % NZ-amine and 0.2 % dextrin to OD_600_ of 0.3–0.4. From each culture, 5 ml was taken and stressed with UV (75 J m^−^²) and further incubated at 75 °C for 3 h. Cell pellets were collected from 2 ml of culture by centrifuging at 5000 ***g*** for 20 min and stored at −20 °C. Cells were lysed in Z buffer (pH 7) (10 mM KCl, 1 mM MgSO_4_, 60 mM Na_2_HPO_4_ and 40 mM NaH_2_PO_4_) supplemented with 0.5 % Triton X-100 to a theoretical OD_600_ of 3.2.

The assay was performed in a 96-well plate, and each reaction contained 25 µl cell lysate, 175 µl Z buffer and 10 µl ONPG solution (40 µM). The assay was carried out at 42 °C for 2–3 h. The yields of ortho-nitrophenol were measured at 410 nm in a microplate reader (CLARIOstar, BMG Labtech).

In parallel with the assay, the protein concentration of the samples was determined by BCA (bicinchoninic acid) kit following the manufacturer’ s protocol.

ONPG activity was calculated using the following formula:

$$x=\frac{60000\times \left[(\text{A}410\left(\text{t}2-\text{t}1\right)-autolysisat410nm\left(t2-t1\right)\right]\times 7}{Time\times Volumeofsample\times Concentrationofprotein}$$

*x* is the β-galactosidase activity in modified Miller units, time is expressed in seconds, the volume of the sample is expressed in millilitres and the concentration of protein is expressed in milligrams per millilitre [38]. The factor 7 was determined as the correction factor for the activity of β-galactosidase at 75 °C under normal growth conditions instead of 42 °C, the maximum temperature at which the assay could be performed [39].

### Construction of substitution promoter strains

All mutants were obtained using the ‘pop-in’, ‘pop-out’ method described previously [37]. First, a background strain was created by deleting 321 bp upstream of *upsX* and 1873 bp of the *upsX* gene, resulting in MW1100. The upstream and downstream regions of the substitution in the target promoters of *upsX* and *upsE (saci_1494)* were amplified by specific overlap primers pairs (Table 2). These regions were connected by overlap PCR using the outside primers 5555/5560. The PCR product was purified and cloned into plasmid pSVA406 by ApaI and PstI, resulting in the substitution plasmids pSVA3625 (for the *upsX* promoter) and pSVA3649 (for the *upsE* promoter). Plasmid transformation to MW1100 and mutant screening was performed as described previously [37]. The resulting strain MW1101 has the substitution of four nucleotides (ATTT) in the *upsX* promoter and MW1103 carrying the substitution of six nucleotides (ACTTTC) in the *upsE* promoter.

### Quantitative RT-PCR

To compare the expression of genes of the *ups* operon before and 3 h after UV induction, RNAs were isolated from 10 ml culture at an OD_600_ of 0.4 using Trizol (Sigma) followed by DNAse I treatment. cDNAs were synthesized using of the First Strand cDNA synthesis Kit (Thermo Scientific), following the manufacturer’ s manual.

qRT-PCR was performed using Maxima SYBR green master mix in a Rotor-Gene Q qPCR machine (Qiagen). Gene-specific primer sets were used for following genes: *upsX (saci_1493)*, *upsE (saci_1494)* and *upsA (saci_1496)* (Table 1). As control, a pair of primers 1480/1481 was used for the housekeeping gene *secY.* The threshold cycle (CT) values obtained were used to compare the non-UV-induced expression to the UV-induced expression of the tested genes. Furthermore, expression levels of *ups* genes of MW001 and *upsX* promoter mutants were compared. Differences in expression are displayed as log2-fold.

### Bioinformatic analyses

To create the sequence logo, 80 bp (from the TSS) of the promoter sequences of *upsX (saci_1493)*, *upsE (saci_1494)*, *upsA (saci_1496)*, *tfb3 (saci_0665)*, *saci_0951, saci_1225* and *saci_1302* were aligned using T-coffee [40] and depicted by WebLogo [41]. The same was done with the respective homologous genes of *Sulfolobus tokodaii* 7, *Sulfolobus solfataricus* P2 and *Sulfolobus islandicus* REY15.

Furthermore, a manual search using Clone Manager was performed to identify genes in the *S. acidocaldarius* genome harbouring the motif A(N)TTTC locating from −30 to −81 bp from their respective start codon. Genes harbouring the A(N)TTTC motif in their promoter region were compared to the list of genes that are regulated upon UV stress [29, 30] (Table S1, available in the online Supplementary Material).

### DNA transfer assay

DNA transfer assays between auxotrophic (Pyr^-^) *S. acidocaldarius* cells were performed as described previously [31]. The background strains MW001 [311 bp deletion (nt 91–412 in *pyrE*)] and JDS22 [22 bp deletion (nt 16–38 in *pyrE*)] were mixed to obtain prototrophic colonies (Pyr^+^). The *upsX* promoter substitution mutant (P*_upsX_*_sub_) was made in the MW001 background as described above. Liquid cultures were grown at 75 °C to an OD_600_ of 0.5–0.6, harvested and subsequently concentrated to an OD_600_ of 1. One half of each culture was UV treated as described above. Cells were mixed (1 ml per culture) and mixtures were incubated for 3 h at 75 °C in 24-well plates. To obtain prototrophic colonies, 200 µl of each mixture was spread on plates lacking uracil and incubated for 6 days at 75 °C.

## Results

### Determination of the minimal active size of the *upsX* promoter

**Fig. 1.**
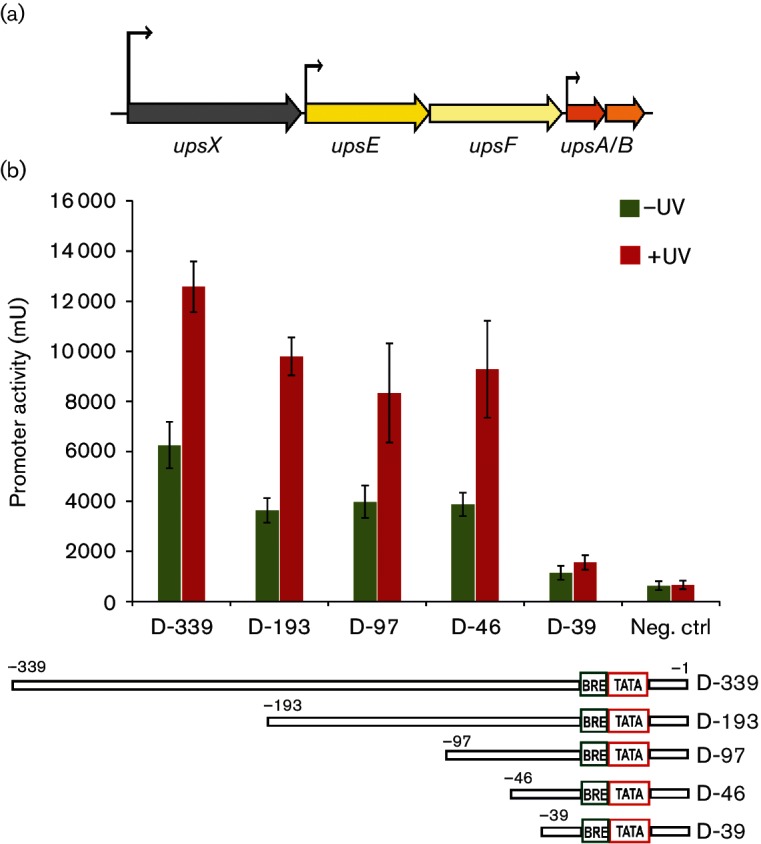
Defining the minimal size of the *upsX* promoter by promoter deletion analysis. (a) Schematic overview of the *ups* operon of *S. acidocaldarius*. The cluster encodes a protein of unknown function, UpsX (Saci_1493), a secretion ATPase UpsE (Saci_1494), an integral membrane protein, UpsF (Saci_1495) and two pilin subunits, UpsA and B (Saci_1496 and Saci_1497, respectively). The transcription start sites are indicated by black arrows. (b) Upper panel: specific β-galactosidase activity from the reporter plasmids containing the truncated *upsX* promoters assayed under non-UV conditions and 3 h after UV treatment. Negative control (Neg. ctrl) is the pCMal LacS plasmid from which the *malE* promoter was removed. Lower panel: truncation of *upsX* promoters in the reporter plasmids. The numbers above each construct indicate the 5′ end of each promoter fragment in respect to the transcription start site.

In recent years, *Sulfolobus* promoters such as the *araS* promoter, the *tf55a* promoter and the *malE* promoter have been investigated using *in vivo* reporter gene experiments [25, 38, 42]. Although *Sulfolobales* display a clear transcriptional response to UV stress, the underlying regulatory processes are not well understood. Deep sequencing data of *S. acidocaldarius* suggested a primary TSS in front of *upsX*, the first gene of the *ups* operon (Fig. 1a), which was found to be highly upregulated upon UV stress [29, 30, 35].

In order to characterize the *upsX* promoter of *S. acidocaldarius*, we defined the minimal size of the *upsX* promoter using the reporter plasmid pcMalLacS [36]. To that end, the *malE* promoter was replaced by different truncated versions of the *upsX* upstream region ranging from −339 bp to −39 bp upstream of the TSS (Fig. 1b). The LacS activity produced from each promoter fragment before and 3 h after UV treatment was determined (Fig. 1b). Expression of LacS under control of the longest *upsX* upstream region (D-339) increased up to twofold after UV stress (Fig. 1b). Similarly, the promoter truncation constructs down to D-46 (D-193, D-97 and D-46) showed the same activation of the promoter after UV light exposure. However, deletion of another seven nucleotides (D-39) abolished the *upsX* promoter activity prior to and after UV treatment, respectively. This indicates that the region from −46 to −39 (5′-GTATTTTC-3′) is required for *upsX* promoter activity. We can therefore conclude that the minimal active size of the *upsX* promoter is between 39 and 46 bp upstream of the TSS.

### Investigation of the region from −46 to −39 in the *upsX* promoter

**Fig. 2.**
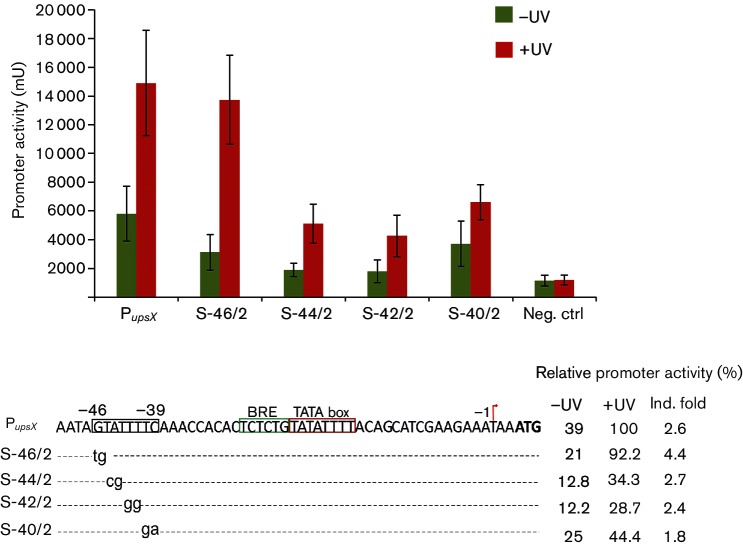
Investigation of the region from −46 to −39 of the *upsX* promoter by promoter substitution analysis. Upper panel: specific *β*-galactosidase activity of the reporter plasmids containing the mutated promoter. Negative control (Neg. ctrl) was the pCmalLacS plasmid of which the *malE* promoter was removed. Lower panel: the construction of the substituted promoters and the relative promoter activity of each construction in comparison to the *upsX* promoter 3 h after UV stress. P*_upsX_* is the longest fragment of *upsX* promoter (D-339 in Fig. 1), others are promoter mutants with information about the location and number of nucleotides which were replaced. Substitutions are indicated by lower case letters and dashed lines representing identical nucleotides. On the right: relative promoter activity in comparison to the *upsX* promoter after UV induction.

To determine the exact sequence in the −46 to −39 region upstream of *upsX*, which is important for regulation, we performed a substitution analysis within this sequence using the LacS reporter gene assay (Fig. 2). Therefore we replaced two nucleotides at a time by their opposite purine/pyrimidine. The replacement of the first two nucleotides of the 5′-(46)-GTATTTTC-(39)-3′ sequence (S-46/2) did not result in any effects on the activity of the promoter in response to UV stress. Substituting the next six nucleotides (from −44 to −39), on the other hand, significantly reduced the strength of the promoter in comparison to that of the native one after UV stress (Fig. 2). The activity of the promoter was reduced to 34 % when the −44 and −45 nucleotides (S-44/2) were exchanged (AT to CG). Substitution of the next two nucleotides (S-42/2: TT to GG) resulted in only 28 % activity in comparison to the native promoter. Substitution of the last two nucleotides (S-39/2) (TC to GA) showed a reduced activity of 55 % compared to the full-length *upsX* promoter. This suggests that the six nucleotides from −44 to −39 (5′-ATTTTC-3′) are crucial for the activity of the *upsX* promoter.

### Identification of a conserved non-palindromic sequence in promoters of *S. acidocaldarius* and *S. tokodaii*

**Fig. 3.**
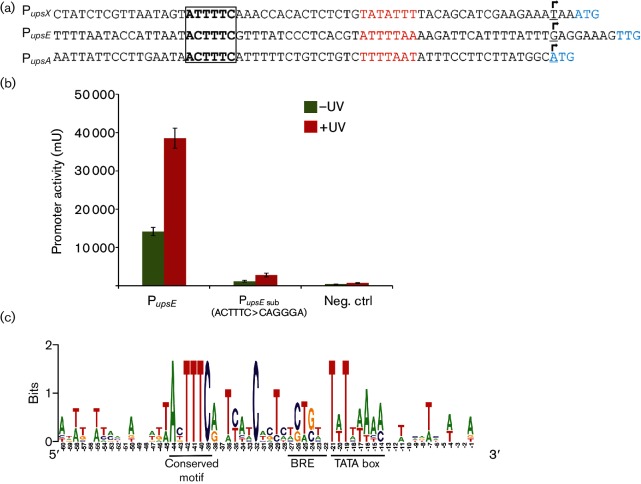
(a) Alignment of promoters of *upsX*, *upsE* and *upsA*; the hexanucleotide motif is boxed, the TATA boxes are highlighted in red; the transcription start sites are underlined with arrows above, and the start codons are shown in cyan. (b) Specific *β*-galactosidase activity of the plasmid containing the substituted promoter of *upsE* assayed without and 3 h after UV stress. The original *upsE* promoter (P*_upsE_*) was used as a positive control. Negative control is the pCmalLacS, from which the *malE* promoter was removed. (c) Conservation of promoter elements in UV-inducible genes in *S. acidocaldarius*: the TATA box, BRE and the hexanucleotide motif are marked with bars below the logo sequence. Promoters of *upsX*, *upsE*, *upsA*, *tfb3*, *saci_1302*, *saci_1225* and *saci_0951* were used to create the sequence logo.

Transcriptome analysis of *S. acidocaldarius* revealed a secondary TSS in front of *upsE* and *upsA*, suggesting that these might have their own promoters [35]. Interestingly, both upstream regions of *upsE* and *upsA* exhibited a similar motif as identified in the *upsX* promoter (5′-ACTTTC-3′) (Fig. 3a). When we replaced this sequence upstream of the BRE site of *upsE* promoter (5′-ACTTTC-3′) by (5′-CAGGGA-3′), its promoter activity was completely abolished (Fig. 3b). Additionally, we found that the promoters of *tfb3*, *saci_0951*, *saci_1225* and *saci_1302* harbour a similar motif. These genes were among the most highly upregulated genes after UV stress [30]. Using the promoter sequences of *upsX*, *upsE*, *upsA*, *tfb3*, *saci_0951*, *saci_1225* and *saci_1302*, we generated a sequence logo presenting a well-conserved, non-palindromic, hexanucleotide motif located from −39 to −44 (5′-A(N)TTTC-3′) from the respective TSSs (Fig. 3c).

Furthermore, we searched the *S. acidocaldarius* genome for additional promoter regions harbouring the 5′-A(N)TTTC-3′ motif located between −80 and −30 bps from the start codon. In total, we found 214 genes, from which 83 were shown to be regulated upon UV stress in either *S. solfataricus* or *S. acidocaldarius* [29, 30] (Table S1). Among these 83 genes, we found *herA* (*saci_0053)* and the cell division control protein encoding *cdc6-2* (*saci_0903)*, two genes that are both upregulated upon UV stress. Among genes that are downregulated upon UV stress, we found those encoding 50S ribosomal proteins and the subunit A1 of the RNA polymerase. Among the non-regulated genes, we found *saci_0669* encoding Urm1/SAMP, an ubiquitin-related modifier. Modification by Umr1, known as urmylation, acts as a signal for substrate recognition by the archaeal proteasome to degrade native and dysfunctional proteins [43].

When searching the genome of the related *S. tokodaii*, the same motif was found in promoters of *STK_13960*, *STK_13970*, *STK_13990*, *STK_16160* and *STK_05280*, which are homologues of *upsX*, *upsE*, *upsA*, *saci_1302* and *saci_0951*, respectively (Fig. S1a). We also searched for the motif in the promoters of the gene homologues of *S. solfataricus* and *S. islandicus*, but the motif is conserved only in a subset of the promoters (Fig. S1b–d). For instance, the orthologue of *upsE*, *SSO0120*, does not harbour the hexanucleotide motif in its promoter region, whereas the motif can be found in the promoter of *SiRe_1879* (Fig. S1d). Interestingly, the promoters of all tested *tfb3* orthologues harbour the motif, suggesting that the regulation of *tfb3* is similar in these species (Fig. S1b). The conservation of the motif in promoters of UV-inducible genes in *S. acidocaldarius*, *S. tokodaii*, *S. solfataricus* and *S. islandicus* suggests a similar regulatory mechanism in these species.

### *In vivo* characterization of the *upsX-* and *upsE-* promoter substitution strains

**Fig. 4.**
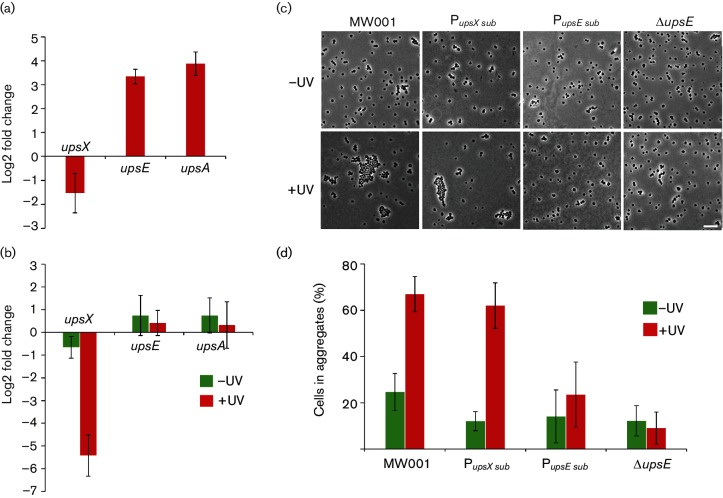
The role of the 5′-A(N)TTTC-3′ motif in transcription of genes in the *ups* operon. (a) Transcription levels of *upsX*, *upsE* and *upsA* 3 h after UV stress in the *upsX* promoter substitution strain (P*_upsX_*_sub_ strain). Change in transcription levels of genes 3 h after UV treatment (75 J m^−^²) was measured by qRT-PCR. Differences are displayed as log2-fold changes. (b) Transcription levels of *upsX*, *upsE* and *upsA* in the P*_upsX_*_sub_ strain in comparison to that of MW001. Changes in the transcription levels of genes were measured by qRT-PCR. Differences are displayed as log2-fold changes. (c) Aggregation assay with the *upsX* and *upsE* substitution promoter strains (P*_upsX_*_sub_ and P*_upsE_*_sub_, respectively). Non-UV treated cells (−UV) and cells 3 h after induction with UV light (75 J m^−2^) (+UV) were visualized using phase-contrast microscopy. MW001 was used as a positive control and Δ*upsE* as a negative control (scale bar, 10 µm). (d) Quantification of cellular aggregation of non-UV-treated cells (−UV) and cells 3 h after UV treatment (+UV). Single and aggregated cells (*n*>3) were counted. Average numbers of cells in aggregates were calculated from three individual experiments.

Our gene fusion reporter assays suggested an important role of the conserved motif in the *upsX* and *upsE* promoters (Figs 2 and3). To study this motif endogenously, a substitution of four nucleotides (5′-ATTT-3′ to 5′-CGGG-3′) of the motif was created in the promoter of *upsX* and a substitution of six nucleotides (5′-ACTTTTC-3′ to 5′-CAGGGA-3′) was introduced in the *upsE* promoter in the MW001 genome. The transcription levels of *upsX*, *upsE* and *upsA* were checked 3 h after UV stress in the *upsX* substitution promoter strain (P*_upsX_* sub-strain). As expected, no upregulation of *upsX* was observed, whereas *upsE* and *upsA* were still highly upregulated (Fig. 4a). In addition, the transcription levels of *upsX*, *upsE* and *upsA* in the P*_upsX_*_sub_ strain were compared to the transcription of these genes in the wild-type (Fig. 4b). Before UV induction, the overall transcription of *upsX* was similar as that of the wild-type. However, after UV stress, the transcription levels of *upsX* were greatly reduced (more than five log2-fold). The transcription levels of *upsE* and *upsA* on the other hand showed no differences from those of the wild-type (Fig. 4b). All the data suggest that the motif has an effect on the expression of UpsX before and after UV treatment.

It has previously been shown that UpsE is essential for ups pili formation and cellular aggregation, while UpsX does not play a role in these processes [34]. As shown in our aggregation assays (Fig. 4c, d), cells from the P*_upsX_*_sub_ strain were still able to form cellular aggregates like the wild-type MW001. However, the P*_upsE_*_sub_ strain was not able to form cellular aggregates similar to the *upsE* deletion strain (Fig. 4c, d). These results indicate that expression of *upsE* after UV stress was effected by the substitution of the motif in its promoter, which subsequently resulted in no pili formation and therefore no cellular aggregation.

**Fig. 5.**
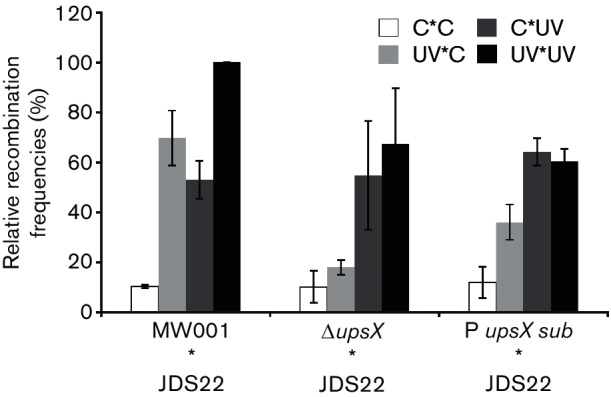
DNA transfer assays using *ups*X deletion mutant as well as *upsX* promoter substitution mutant (P*_upsX_*_sub_). Two different background strains (MW001 and JDS22) contained mutations in the *pyrE* gene (involved in *de novo* synthesis of uracil). Two auxotrophic strains were treated with (UV) or without (C) UV light and mixed and plated on selective media to obtain prototrophic colonies (Pyr+). Bars represent the average of at least three independent mating experiments each. Every experiment was normalized to JDS22 (UV) * MW001 (UV) as 100 %.

Next we tested the DNA transfer capability of the P*_upsX_*_sub_ strain. We expected a reduction in DNA transfer comparable to the phenotype observed when mixing a *upsX* deletion strain with MW001 [34]. The performed DNA transfer assays rely on the selection of prototrophic colonies (*pyrE*+) that contain a restored *pyrE* locus after DNA transfer and homologous recombination. To that end we mixed two different *pyrE* deletion background strains (MW001 and JDS22) as described previously [31]. As expected, we observed fewer prototrophic colonies (*pyr+)* in the mixtures of Δ*upsX* or P*_upsX_*_sub_ with JDS22 compared to the background mixtures MW001xJDS22 when both mating partners were UV treated (UV*UV; mixtures 4, 8 and 12) (Fig. 5). Moreover, when Δ*upsX* or P*_upsX_* strains were treated with UV, but not the background strain JDS22 (UV*C; mixtures 6 and 10, respectively), no significant increase of DNA transfer was observed. This is in contrast to the situation where only MW001 (UV*C, mixture 2) or JDS22 was UV treated (C*UV; mixtures 7 and 11). This again supports the importance of UpsX in DNA transfer in *S. acidocaldarius*.

## Discussion

Upon DNA damage, Sulfolobales exhibit a distinct transcriptional response resulting in the repression of the DNA replication machinery and the increased expression of oxidative stress enzymes and proteins involved in DNA transfer [29, 30]. As part of our aim to elucidate the poorly understood UV-inducible stress response of *S. acidocaldarius,* we investigated the promoter region of *upsX*, a gene highly expressed upon DNA damage. Using the LacS reporter system, we determined the minimal active size of the *upsX* promoter to be 46 bp from the TSS, similar to other known archaeal promoters [25, 44] (Fig. 1b). Within the 46 bp promoter region of *upsX*, we identified upstream of the TATA box and BRE site a non-palindromic, six-nucleotide sequence (5′-ATTTTC-3′) from −44 to −39 as being crucial for the *upsX* promoter (Fig. 2). In addition, the upregulation of *upsX* upon UV stress was eliminated in the P*_upsX_*_sub_ strain in which the four nucleotides 5′-ATTT-3′ (from −44 to −40) were substituted to CGGG (Fig. 4a), suggesting the importance of the upstream motif in the activity of the *upsX* promoter at its native locus. This is supported by the fact that the P*_upsX_*_sub_ strain has a similar reduced DNA transfer capability to the clean *upsX*-deletion strain (Fig. 5, [34]. Furthermore, the substitution of the motif in the *upsX* promoter had no effect on *upsE* and *upsA* transcription levels after UV treatment (Fig. 4a, b), which is in good agreement with the three independent transcription starts of *upsX*, *upsE* and *upsA*. Indeed, we found a similar motif −44 to −38 in front of the *upsE* and *upsA* promoters, which only differs at the second position (5′-A(C)TTTC-3′) (Fig. 3a). As expected, the P*_upsE_*_sub_ strain was unable to form cellular aggregates upon UV light treatment, like the *upsE* deletion strain, again underlining the importance of the upstream motif in the transcription of UV-regulated genes (Fig. 4c, d).

Most notably, the upstream motif of the three *ups* promoters is not palindromic. Palindromic sequences are usually important for base-specific interactions in the major groove of the DNA, either with the recognition α-helix of an HTH motif or with the β-sheet face of an RHH motif of the specific regulator [45, 46]. Nevertheless, in some cases the binding sites of regulators are not always perfectly palindromic. For instance, the 24 bp binding site of the heat shock regulator Phr *of Pyrococcus furiosus* is only palindromic for 3 bp of its 5′ prime and 3′ prime ends, whereas the rest of the motif is non-palindromic [18]. Furthermore, the activator PF1088 binds to a non-palindromic sequence in the promoter of *pf1089* in *P. furiosus* to stimulate the recruitment of TFB to the *pf1089* operon [20]. In addition, activation of transcription mediated by PF1088 was strongly dependent on an imperfect BRE site. A similar mode of action was also proposed for other regulators binding to a motif upstream of weak BRE sites, including the unknown activator of the Ara Box of *S. solfataricus* [22] and *S. islandicus* [25], but also ArnR-binding to the ArnR box 1 in *S. solfataricus* [23]. As was shown in previous studies, a strong BRE site of *Sulfolobus* promoters contains an A at positon −3 and A/G at −6 in respect to the TATA box [47, 48]. There is neither a conserved A at −3 nor are A/G at −6 highly conserved in the BRE sites of *ups* promoters, suggesting that these BRE sites are weak (Fig. 3c). Given the fact that the *ups* promoters also contain a weak BRE site, we speculate that an unknown regulator binds to the upstream 5′-A(N)TTTC-3′ motif, which helps to recruit a TFB to these promoters. It was previously shown that after UV stress *tfb3* is highly induced in *S. solfataricus* and *S. acidocaldarius* [29, 30]. This basal transcription factor lacks a DNA-binding domain, but was shown to interact with the RNAP. Furthermore, TFB3 was shown to stimulate transcription of promoters *in vitro* in the presence of TFB1-TBP-DNA [49]. We speculate that TFB3 is recruited to the promoter by binding to the unknown regulator that is bound to the upstream 5′-A(N)TTTC-3′ motif. As suggested in earlier studies, TFB3 probably serves as a molecular bridge between the RNAP and the TFB1-TBP-DNA complex, thus enhancing the transcription of genes [49].

Interestingly, the upstream motif is not only restricted to the *ups* and *tfb3* promoters, but was also found in the promoter region of 214 genes in total (Table S1). This might be an overestimate since a six-nucleotide, AT-rich sequence can be frequently found in the genome of *S. acidocaldarius*. However, among these we found genes encoding the DNA repair protein HerA and the DNA replication control protein Cdc6-2. These two genes are significantly upregulated upon UV stress, similar to the *ups* genes [30, 50]. Both HerA and Cdc6-2 are known to be key factors in maintaining genome integrity, due to their involvement in homologous recombination and inhibition of DNA replication, respectively [51, 52]. Additionally, we found some genes in our analysis that were slightly repressed following UV treatment, like the RNA polymerase subunit rpoA1 or some 50S ribosomal proteins (Saci_0576, Saci_0584). This finding suggests that the motif might act as a recognition site not only for an activator, but also for a repressor. Interestingly, the majority of the genes (131) that harbour the upstream motif were not found to be regulated by UV stress by more than 0.5 log2-fold (Table S1, [29, 30]). However, this high number might also be an overestimate, because the previous microarray data did not identify all genes that are induced following UV stress, including *upsX* [30].

Furthermore, some genes whose transcription is altered upon UV stress do not possess the upstream motif in their respective promoter region. These include the *ced* loci whose gene products play an essential role in DNA transport after UV irradiation [31]. Thus it seems that these genes are under the control of another regulator. One candidate might be the iron-dependent regulator Saci_0161, which is upregulated upon UV stress and was identified in our study to harbour the 5′-A(N)TTTC-3′ motif in its promoter region (Table S1; [30]).

Taken together, we identified a non-palindromic hexanucleotide sequence upstream of the UV-inducible promoters of the *ups* operon. Substitution of this upstream motif leads to the abolishment in cellular aggregation and reduced DNA transfer, respectively. Moreover, the upstream motif is found in a large number of genes that are either up- or downregulated. Future studies need to (1) determine the role of the upstream motif in the repression of genes and (2) confirm our model of the expression of genes (see above) by identifying and characterizing the regulator that binds to the upstream motif. The presence of the motif in promoters of other *Sulfolobus* species suggest a common regulatory mechanism in these organisms.

## Supplementary Data

596 Supplementary File 1
