# Supplementary material for: A conserved hexanucleotide motif is important in UV-inducible promoters in Sulfolobus acidocaldarius
Source: Microbiology (Reading). 2017 May 3;163(5):778–88. doi: 10.1099/mic.0.000455 (PMC5817253; doi:10.1099/mic.0.000455)
Supplement: Supplementary File 1 [file mic-163-778-s001.pdf]

Supplementary Material for

A conserved hexanucleotide motif is important in UV inducible promoters in *Sulfolobus acidocaldarius*

Thuong Ngoc Le, Alexander Wagner, Sonja-Verena Albers*

Molecular Biology of Archaea, Institute of Biology II - Microbiology, University of Freiburg, Schaenzlestrasse 1, 79104, Freiburg, Germany

Running head: A motif in UV inducible promoter in *Sulfolobus acidocaldarius*

*Corresponding author: S.V. Albers, e-mail: sonja.albers@biologie.uni-freiburg.de, tel.: +49(0)761 203 2630

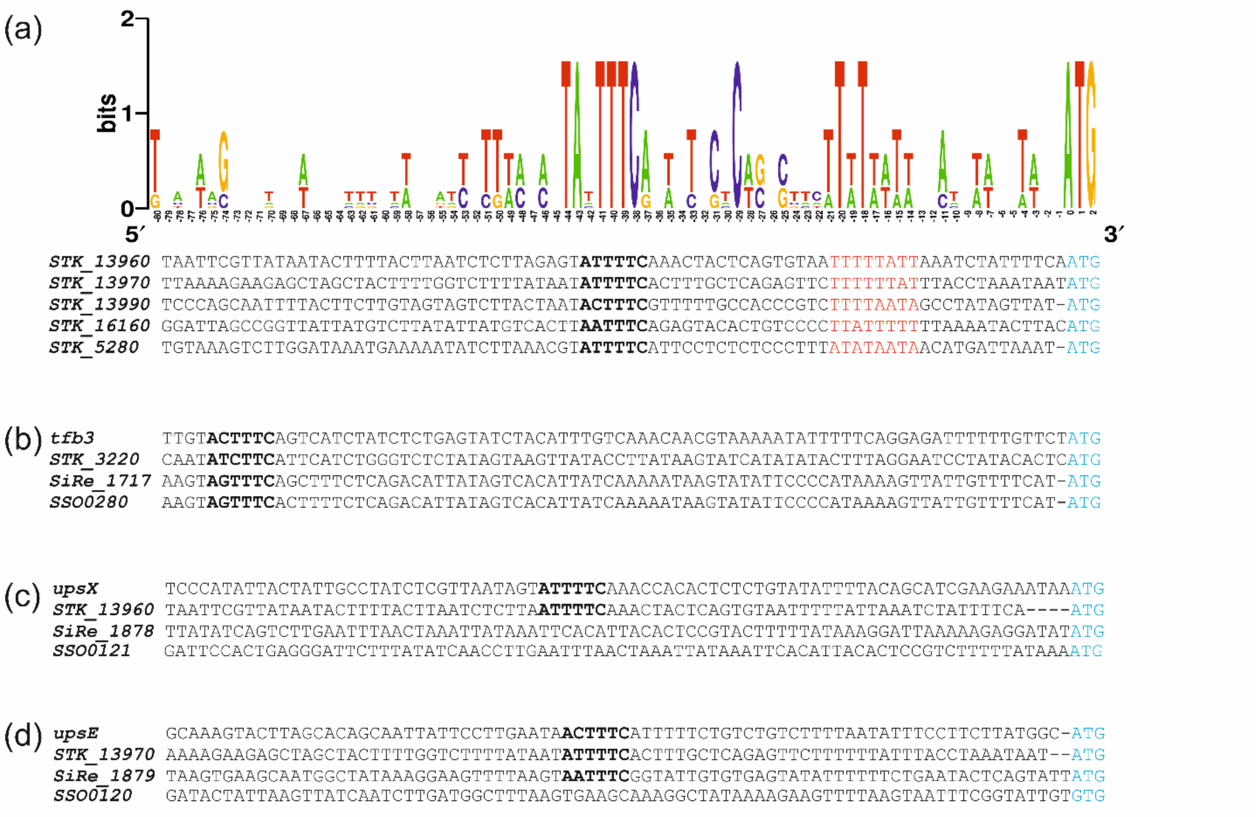

Supplementary Figure 1:

(a): Conservation of promoter elements in UV inducible genes in *S. tokodaii:* the conserved motif, the TATA box, the start codon are marked with black, red and cyan letters, respectively. Promoters of *STK-13960, STK_13970, STK_13990, STK_16160,* and *STK_05280* were used to create the sequence logo.

(b), (c), (d): Promoter alignments of of *tfb3, upsX, upsE* homologs in *S. solfataricus P2, S. tokodaii 7, S. islandicus REY15.* The conserved motif was marked with bold letters.
